# Supplementary material for: Intergenerational transmission of polygenic predisposition for neuropsychiatric traits on emotional and behavioural difficulties in childhood
Source: Nat Commun. 2025 Mar 18;16:2674. doi: 10.1038/s41467-025-57694-w (PMC11920414; doi:10.1038/s41467-025-57694-w)
Supplement: Supplementary file 1 — Supplementary Information [file 41467_2025_57694_MOESM1_ESM.pdf]

Supplementary Information

Intergenerational transmission of polygenic predisposition for neuropsychiatric traits on emotional and behavioural difficulties in childhood

Allegrini, A. G. [1,2#], Hannigan, L.J. [3,4,5], Frach, L.[1,6], Barkhuizen, W.[1], Baldwin J. R.[1,2], Andreassen, O.A. [7], Bragantini, D. [3,4], Hegemann, L. [3,4,8], Havdahl, A. [3,4,9]*, Pingault J-B. [1,2]*

# = Corresponding author

* = These authors jointly supervised this work

Affiliations:

1. Department of Clinical, Educational and Health Psychology, Division of Psychology and Language Sciences, University College London, London, UK.
2. Social, Genetic and Developmental Psychiatry Centre, Institute of Psychiatry, Psychology and Neuroscience, King's College London, London, UK.
3. Research Department, Lovisenberg Diaconal Hospital, Oslo, Norway
4. PsychGen Center for Genetic Epidemiology and Mental Health, Norwegian Institute of Public Health, Oslo, Norway
5. MRC Integrative Epidemiology Unit (IEU), University of Bristol, Bristol, United Kingdom
6. Institute of Human Genetics, University of Bonn, School of Medicine & University Hospital Bonn, Bonn, Germany
7. NORMENT Centre, Institute of Clinical Medicine, University of Oslo and Division of Mental Health and Addiction, Oslo University Hospital, Oslo, Norway
8. Department of Psychology, University of Oslo, Oslo, Norway
9.PROMENTA Research Centre, Department of Psychology, University of Oslo, Oslo, Norway

Contents

Model comparison and suitability - page 3

Power simulations - page 4

Symptom- and domain-level heterogeneity - page 8

Supplementary figures - page 9

Model comparison and suitability

We fitted a second-order and a symmetric bifactor models, where items of different emotional and behavioural difficulties loaded on specific latent factors, reflecting their corresponding scales. Both models fitted the data well and the bifactor model was favoured over the second-order model based on standard fit indices and nested comparison (supplementary tables S2). However, inspection of the model indicated that this was not the most suitable parameterization for our analyses. First, several inconsistencies emerged in p-factor loadings. One anxiety item from the SCARED questionnaire corresponding to “my child is shy” had a nonsignificant negative loading on the p-factor (beta = -.036, se = .030, p =.220). Second, two depression items from the SFMQ corresponding to “child is very restless” and “finds it hard to think or concentrate” loaded poorly on the depression factor, but strongly on the p-factor (standardized loadings respectively: .083 and .103 vs .655 and .782). Similarly, four hyperactivity items (hyp128, hyp129, hyp130, hyp131; see Table S1) loaded poorly on the hyperactivity factor (standardized weights < .3), but more heavily on the p-factor (~.7 to ~.8, Tables S3). Finally, the variance of the hyperactivity factor was near zero. Table S3 reports standardized loadings and factor variances for the bifactor and second-order models. Taken together results suggest a partially collapsing factor, with the general domain reflecting hyperactivity rather than a shared psychopathology dimension across traits. As an additional caveat, we found that the bifactor model presents a number of challenges when employed in multiple indicators multiple causes (MIMIC) approaches such as our planned analyses. In particular, attempting to explain either the general or specific factors from a bifactor model with external predictors can lead to biased parameter estimates, as documented elsewhere [1]. In conclusion the symmetrical bifactor model was not suitable for the data at hand (see also [2] for further discussion), hence we carried forward in analyses the second order model.

Power simulations

We performed power simulations for a second-order multiple indicators multiple causes (MIMIC) model, to investigate our power to detect the effects of parent-offspring polygenic scores (PGS) on the general and specific domains. Our simulations were based on parameter estimates and causal model specified in [3]. We used weights from the base second-order model to simulate data and run power simulations based on a combination of 3 data-generating scenarios involving joint effects of PGS for parents and offspring on either the general or the specific EBP domains. Each of the combinations was tested across fixed parameters for the child and mother PGS (explaining 0.2% and 0.1% of the variance respectively, corresponding to a beta ~ .04, and of beta ~ .03) and three different parameters for the father polygenic score (betas .03, .01, .001), holding constant sample size at N = 15,000 (our maximum sample size). For each data generating mechanisms we tested for joint effects of the parent-offspring PGS over either the general or the specific domains. Power was calculated as the sum of p-values less than $\alpha = 0.05$ divided by the number of iterations, set to 10,000.

Model Setup

Offspring PGS:

$$PGS_c = \frac{1}{2}(PGS_m + PGS_f) + e_{PGS}$$

$$var(e_{PGS}) = 0.5$$

The offspring phenotype is defined as a linear combination of the parents and offspring PGS, this varies depending on the structural model employed in simulations, below:

$$Y_c = b^d PGS_c + b^{i,m} PGS_m + b^{i,f} PGS_f + e_y$$

Where b^d is the direct genetic effect, and $b^{i,m}$ $b^{i,f}$ are the maternal and paternal indirect genetic effects respectively.

We also set mean 0 and variance 1 for all PGS:

$$E(PGS_c) = E(PGS_m) = E(PGS_f) = 0$$

$$var(PGS_c) = var(PGS_m) = var(PGS_f) = 1$$

And assume no assortative mating:

$\mathrm{cov}(PGS_f, PGS_m) = 0$

Measurement model

Y = Λη + ϵ

where Λ is a matrix of regression coefficients of the symptom-level indicators on the corresponding scale-level factors and η = (ODD, CND, HYP, INAT, DEP, ANX).
Foreach scale-level factor the measurement model included equations such as the following:

Oppositional-defiant disorder factor:

$$
\begin{aligned}
odd_1 &= 0.795 \times \text{ODD} + \epsilon_{odd1} \\
odd_2 &= 0.811 \times \text{ODD} + \epsilon_{odd2} \\
odd_3 &= 0.835 \times \text{ODD} + \epsilon_{odd3} \\
odd_4 &= 0.699 \times \text{ODD} + \epsilon_{odd4} \\
odd_5 &= 0.718 \times \text{ODD} + \epsilon_{odd5} \\
odd_6 &= 0.652 \times \text{ODD} + \epsilon_{odd6} \\
odd_7 &= 0.813 \times \text{ODD} + \epsilon_{odd7} \\
odd_8 &= 0.806 \times \text{ODD} + \epsilon_{odd8}
\end{aligned}
$$

Standardized loadings for all indicators are reported in Table S3a.

Structural model

We simulate population models based on the measurement part as described above and three different structural models. These included specifying parent-offspring PGS effects on either 1. the general factor, 2. all the specific factors, and 3. a subset of the specific factors including the lowest and highest weighing domains on the general domain (ANX and HYP respectively), plus a third domain (DEP).

Specifically, the structural model for each factor in the second order MIMIC model is defined

$\eta = B\eta + \Gamma X + \zeta$

where B is a matrix representing the relationships between the latent factors. The regression weights of the first-order scale-level factors η_f = (ODD, CND, HYP, INAT, DEP,

ANX) on the second-order general factor (P) were Β = (0.764, 0.718, 0.837, 0.768, 0.74, 0.369), respectively. For the scale-level specific factors Β = 0. Γ is a matrix of regression weights for the parent-offspring PGS effects on the general or specific factors, and ζ the residuals. For example, for effects of parent-offspring PGS specified over the ODD domain:

$$ODD = .764 \times P + .04 \times PGS_c + .03 \times PGS_m + .01 \times PGS_f + \varepsilon_{odd}$$

The three Γ matrices specified across scenarios involved were as follows:

Population model 1

$$\begin{array}{r} \\ \boldsymbol{P} \\ \boldsymbol{ODD} \\ \boldsymbol{CND} \\ \boldsymbol{HYP} \\ \boldsymbol{INAT} \\ \boldsymbol{DEP} \\ \boldsymbol{ANX} \end{array} \begin{array}{c} \begin{array}{ccc} \boldsymbol{PGSc} & \boldsymbol{PGSm} & \boldsymbol{PGSf} \end{array} \\ \begin{bmatrix} .04 & .03 & \{.001,.01,.03\} \\ 0 & 0 & 0 \\ 0 & 0 & 0 \\ 0 & 0 & 0 \\ 0 & 0 & 0 \\ 0 & 0 & 0 \\ 0 & 0 & 0 \end{bmatrix} \end{array}$$

Population model 2

$$\begin{array}{r} \\ \boldsymbol{P} \\ \boldsymbol{ODD} \\ \boldsymbol{CND} \\ \boldsymbol{HYP} \\ \boldsymbol{INAT} \\ \boldsymbol{DEP} \\ \boldsymbol{ANX} \end{array} \begin{array}{c} \begin{array}{ccc} \boldsymbol{PGSc} & \boldsymbol{PGSm} & \boldsymbol{PGSf} \end{array} \\ \begin{bmatrix} 0 & 0 & 0 \\ .04 & .03 & \{.001,.01,.03\} \\ .04 & .03 & \{.001,.01,.03\} \\ .04 & .03 & \{.001,.01,.03\} \\ .04 & .03 & \{.001,.01,.03\} \\ .04 & .03 & \{.001,.01,.03\} \\ .04 & .03 & \{.001,.01,.03\} \end{bmatrix} \end{array}$$

Population model 3

$$\begin{array}{r} \\ \boldsymbol{P} \\ \boldsymbol{ODD} \\ \boldsymbol{CND} \\ \boldsymbol{HYP} \\ \boldsymbol{INAT} \\ \boldsymbol{DEP} \\ \boldsymbol{ANX} \end{array} \begin{array}{c} \begin{array}{ccc} \boldsymbol{PGSc} & \boldsymbol{PGSm} & \boldsymbol{PGSf} \end{array} \\ \begin{bmatrix} 0 & 0 & 0 \\ 0 & 0 & 0 \\ .0 & 0 & 0 \\ .04 & .03 & \{.001,.01,.03\} \\ 0 & 0 & 0 \\ .04 & .03 & \{.001,.01,.03\} \\ .04 & .03 & \{.001,.01,.03\} \end{bmatrix} \end{array}$$

For corresponding data generating mechanisms (e.g. estimating PGS effects on the P factor in population model 1), we had at least 80% power to detect a direct genetic effect of beta =

.04, and an indirect genetic effect of beta = .03. By contrast, figure S1 shows that if the true data generating mechanism involved effects fully mediated by the general domain, we would be underpowered to detect indirect genetic effects on the specific domains. In particular, the anxiety factor was underpowered for both direct and indirect genetic effects, as expected by the lower load on the general domain. Conversely, if the true data generating mechanism involved only effects on all the specific domains we would also be adequately powered for the general domain (with 80% power to detect an indirect genetic effect as low as beta = .02) Figure S2. However, population model 2 is an unlikely scenario, as it involves equal effects over all specific domains, which is probably unrealistic for any given polygenic score, but is presented here as a proof of concept to show that power is highest for effects on the general domain even in the absence of fully mediated effects. In practice this would also impact a scenario where the true data generating mechanism involves a partial mediation of the PGS effects by the general domain. Finally, results for population model 3 (Figure S3) show that if the true data generating mechanisms involves specific PGS effects to only a subset of domains the gain in power for the general domain is modest.

Symptom- and domain-level heterogeneity

For a PGS to be completely mediated by the common factor, effects do not need to be equal across indicators, but instead should scale in proportion to item loadings on the latent factor [4]. In the case of ADHD, for example, supplementary figure S6 shows beta estimates of the ADHD PGS effects on hyperactivity items vs the loadings of the items on the hyperactivity specific domain. For this domain, where the heterogeneity model was favoured, it can be observed that the ADHD PGS is more important for impulsivity items involved in motion rather than speech (e.g. items 133 and 134 corresponding to "talks excessively" and "Blurts out answers before questions have been completed" respectively). For example, items 135 and 136 corresponding to "difficulties awaiting turn" and "interrupts others" have very similar loadings to items 128 and 129 having to do with "fidgeting" and "leaving the seat when inappropriate", but lower PGS effects. Other examples of item-level heterogeneity were evident for the ADHD and chronic pain PGS on ODD items, where a clear outlier was observed (Figures S7-S8). With this outlier removed, however, item loadings still wouldn't scale in proportion to PGS effects. A second example of heterogeneity is for specific domains involving both direct and indirect genetic effects of the neuroticism PGS (Figure S9). Here the anxiety dimension is an outlier in terms of the child PGS, which have a much higher effect on this domain as a function of loading on the p-factor compared to the rest. In addition, the father and mother PGS contributions are near zero, or negative. As a comparison with these domain and symptom heterogeneity models, figure S10 shows direct and indirect genetic effects for polygenic-P, for which the P-mediated model was favoured.

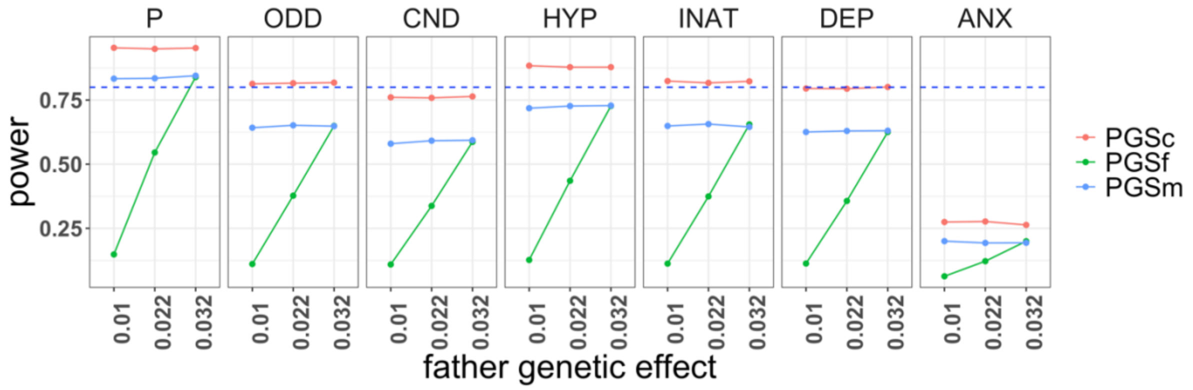

Figure S1. Power analyses for parent offspring polygenic score (PGS) effects over either the general (P) or specific domains based on Population model 1, across 10,000 iterations. Sample size was fixed at N = 15,000. Parameters for the child and mother PGS were fixed to explain 0.2% and 0.1% of the variance respectively, the father PGS was varied across three parameters as shown on the x axis (betas = .03, .01, .001). **Note:** red = estimated offspring PGS effects; Blue = estimated mother PGS effects, Green = estimated father PGS effects.

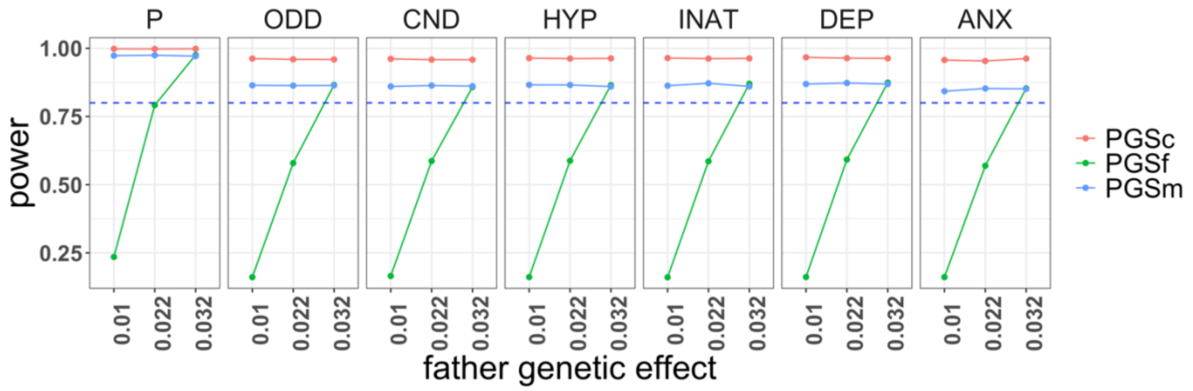

Figure S2. Power analyses for parent offspring polygenic score (PGS) effects over either the general (P) or specific domains based on Population model 2, across 10,000 iterations. Sample size was fixed at N = 15,000. Parameters for the child and mother PGS were fixed to explain 0.2% and 0.1% of the variance respectively, the father PGS was varied across three parameters as shown on the x axis (betas = .03, .01, .001). **Note:** red = estimated offspring PGS effects; Blue = estimated mother PGS effects, Green = estimated father PGS effects.

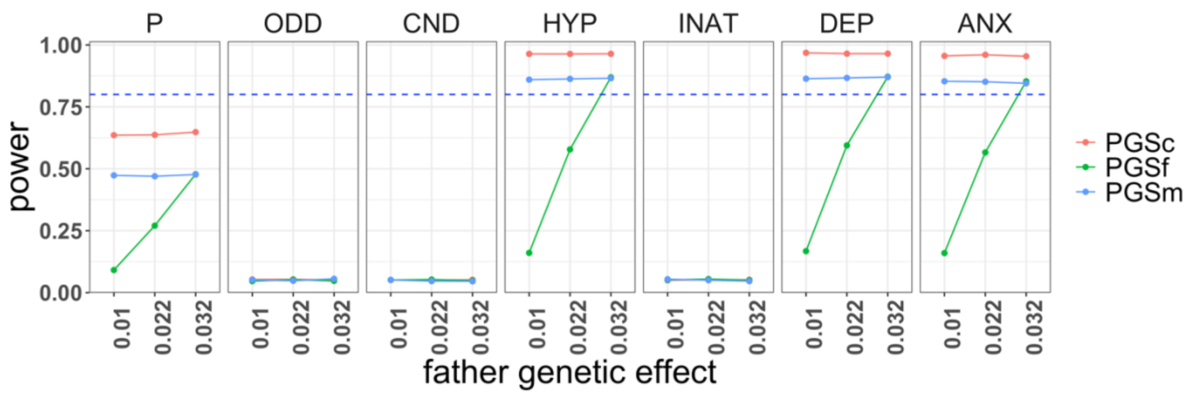

Figure S3. Power analyses for parent offspring polygenic score (PGS) effects over either the general (P) or specific domains based on Population model 3, across 10,000 iterations. Sample size was fixed at N = 15,000. Parameters for the child and mother PGS were fixed to explain 0.2% and 0.1% of the variance respectively, the father PGS was varied across three parameters as shown on the x axis (betas = .03, .01, .001). **Note:** red = estimated offspring PGS effects; Blue = estimated mother PGS effects, Green = estimated father PGS effects.

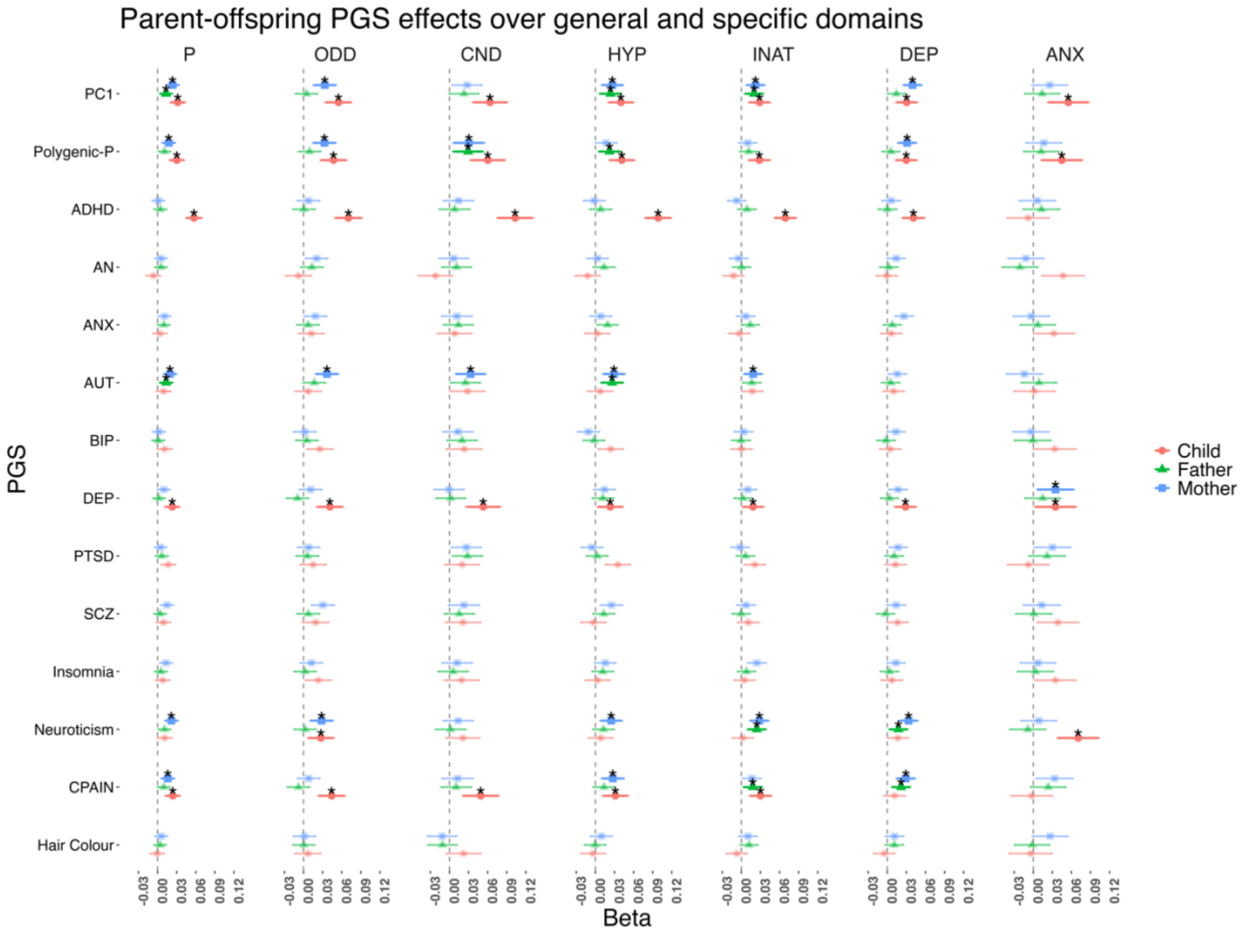

Figure S4. Parent-offspring PGS effects on general and specific emotional and behavioral difficulties domains. Effect sizes and relative confidence intervals for conditional models considering parent and offspring polygenic scores (PGS) effects on the general (P) and specific emotional and behavioural difficulties domains. **Note:** Sample size N = 14,959**.** Point estimates represent beta coefficients and error bars are 95% CIs. ADHD = attention deficit hyperactivity disorder, autism, BIP = bipolar disorder, SCZ = schizophrenia, AN = anorexia nervosa, ANX = anxiety, AUT = autism, PTSD = post-traumatic stress disorder, DEP = broad depression, CPAIN = chronic pain, PC1 = first unrotated principal component of all psychiatric (and related) PGS, Polygenic-P = first unrotated principal component of all psychiatric PGS. **Facets:** ODD = Oppositional Defiant Disorder; CND = Conduct Disorder; HYP = Hyperactivity; INA = Inattention; DEP = Depression; ANX = Anxiety. **Faded**: PGS effect does not survive correction for multiple testing or conditional model not selected over the null model. * = survives correction for multiple testing (Methods).

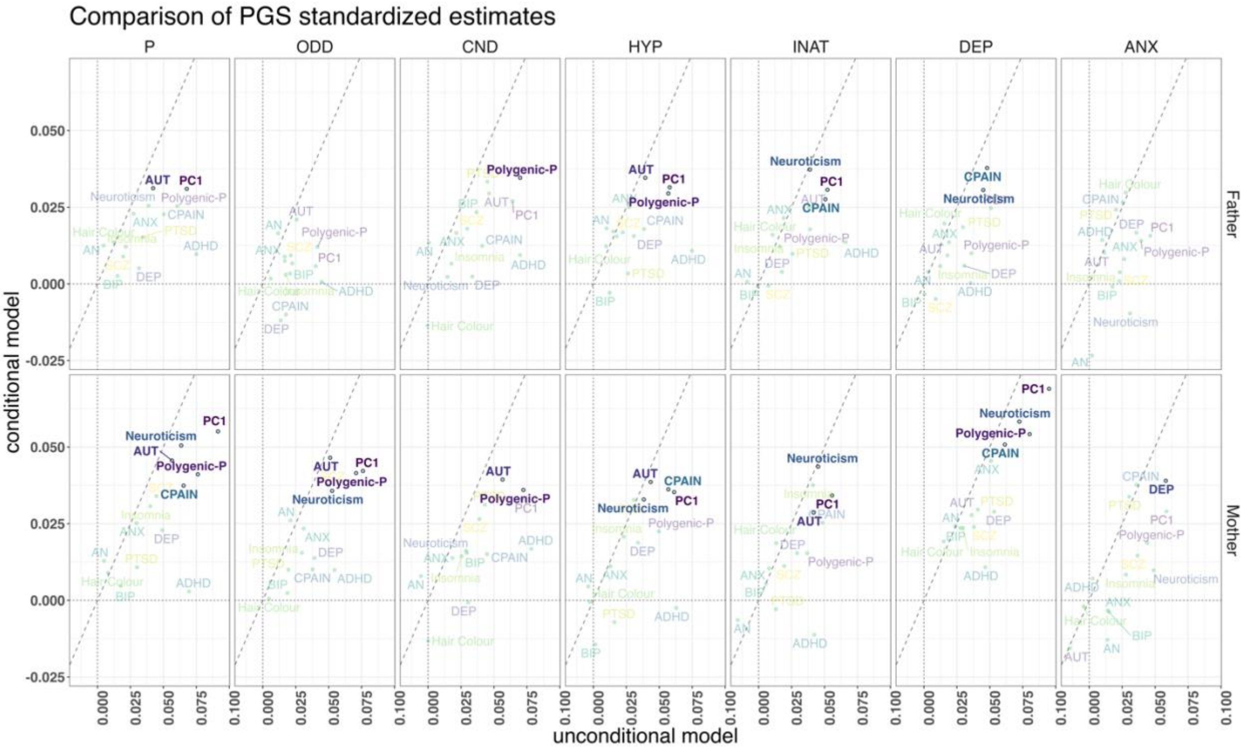

Figure S5. Indirect polygenic scores (PGS) contributions across emotional and behavioural difficulties. Comparison of standardized regression coefficients for offspring PGS effects from conditional to unconditional models, showing relative importance of PGS contributions across domains. **Faded**: does not survive correction for multiple testing/not selected over the null model. **Note:** ADHD = attention deficit hyperactivity disorder, AUT = autism spectrum disorder, BIP = bipolar disorder, SCZ = schizophrenia, AN = anorexia nervosa, ANX = anxiety, PTSD = post-traumatic stress disorder, DEP = broad depression, CPAIN = chronic pain, PC1 = first unrotated principal component of all psychiatric (and related) PGS, PC1 psych = first unrotated principal component of all psychiatric PGS. **Facets:** ODD = Oppositional Defiant Disorder; CND = Conduct Disorder; HYP = Hyperactivity; INA = Inattention; DEP = Depression; ANX = Anxiety.

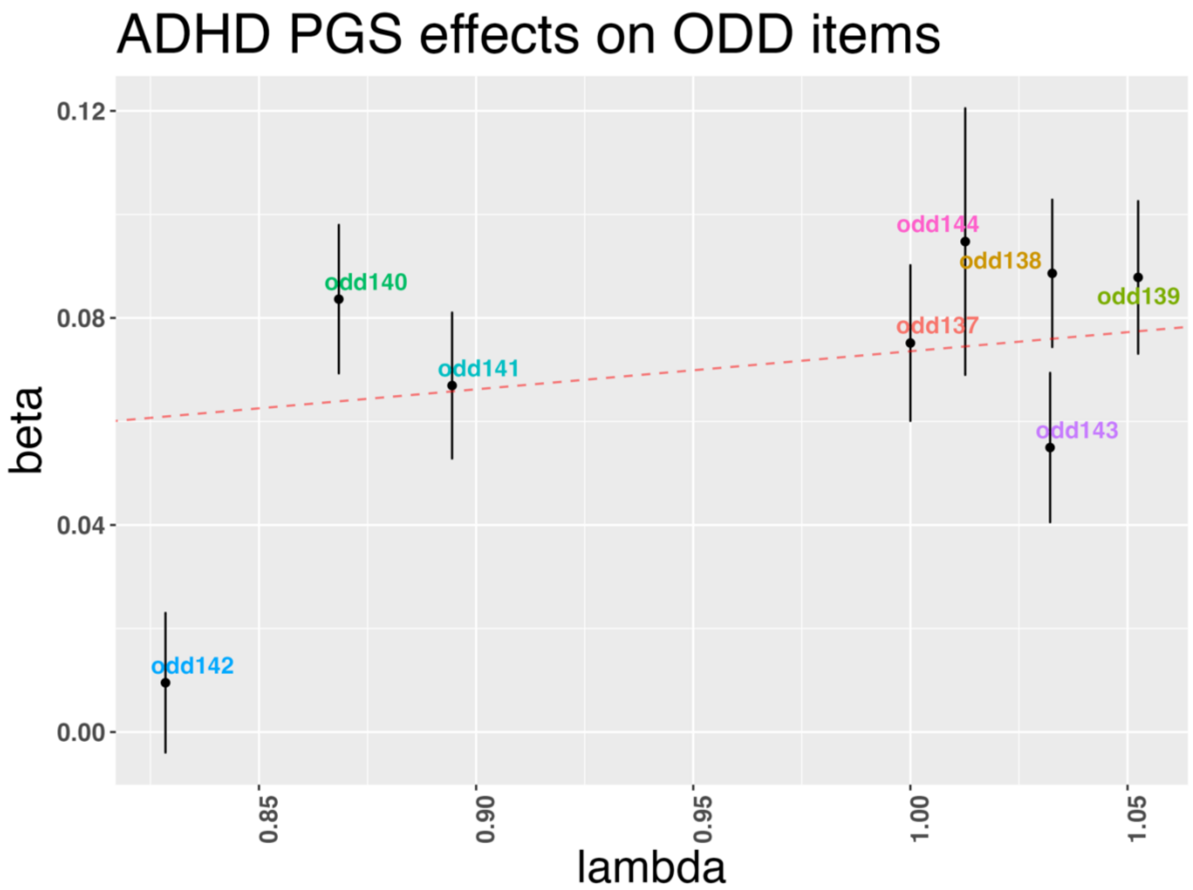

Figure S6. ADHD PGS effects (direct genetic effects) over ODD items against item loadings for the ODD specific domain. Sample size N = 14,959**.** Point estimates represent beta coefficients and error bars are 95% CIs.

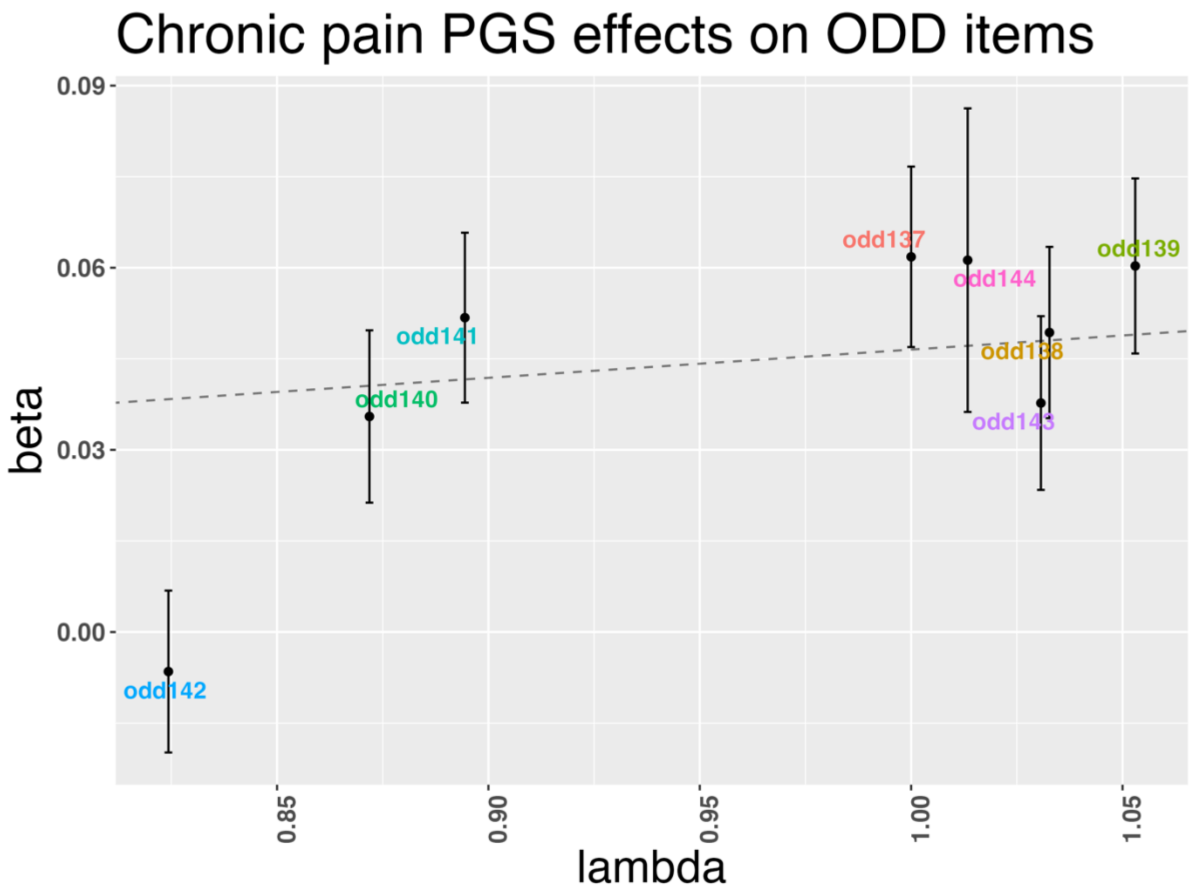

Figure S7. CPAIN PGS effects (direct genetic effects) over ODD items against item loadings for the ODD specific domain. Sample size N = 14,959**.** Point estimates represent beta coefficients and error bars are 95% CIs.

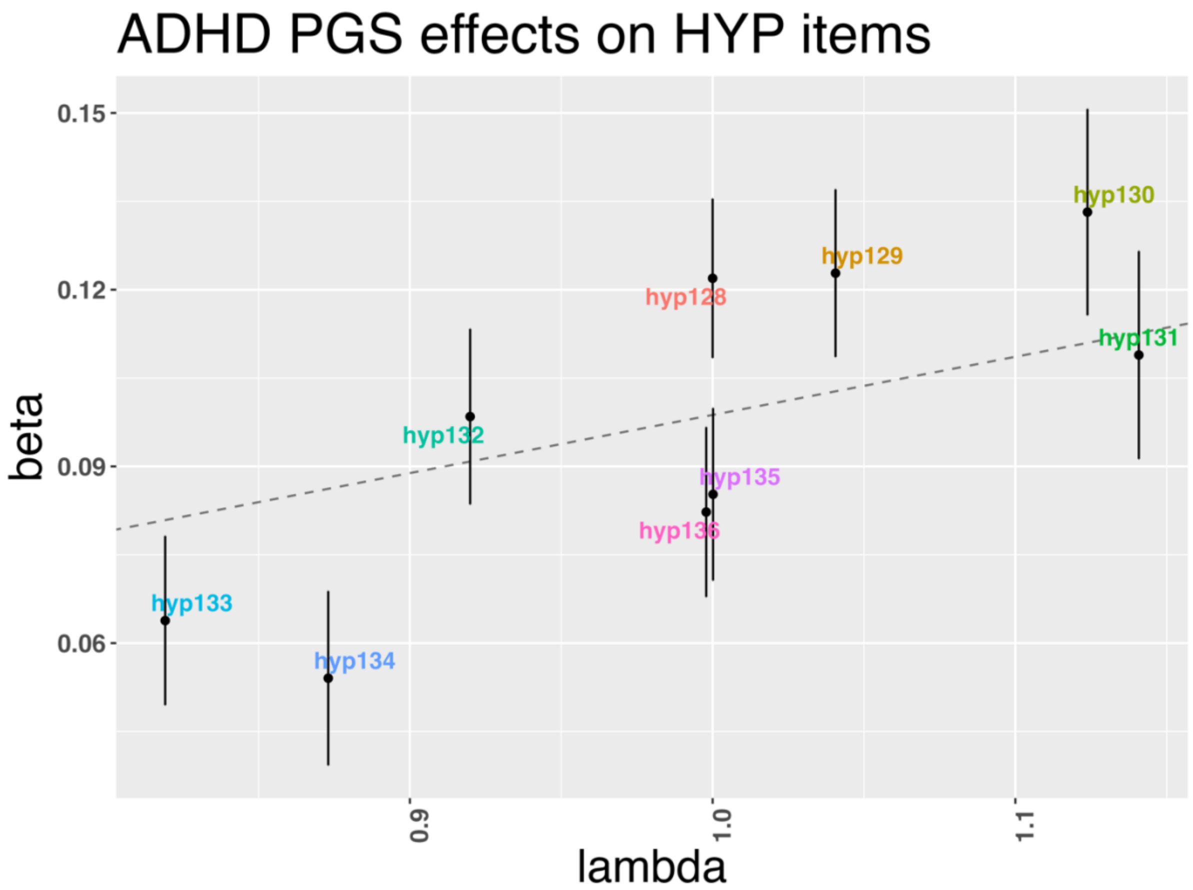

Figure S8. ADHD PGS effects (direct genetic effects) over HYP items against item loadings for the HYP specific domain. Sample size N = 14,959**.** Point estimates represent beta coefficients and error bars are 95% CIs.

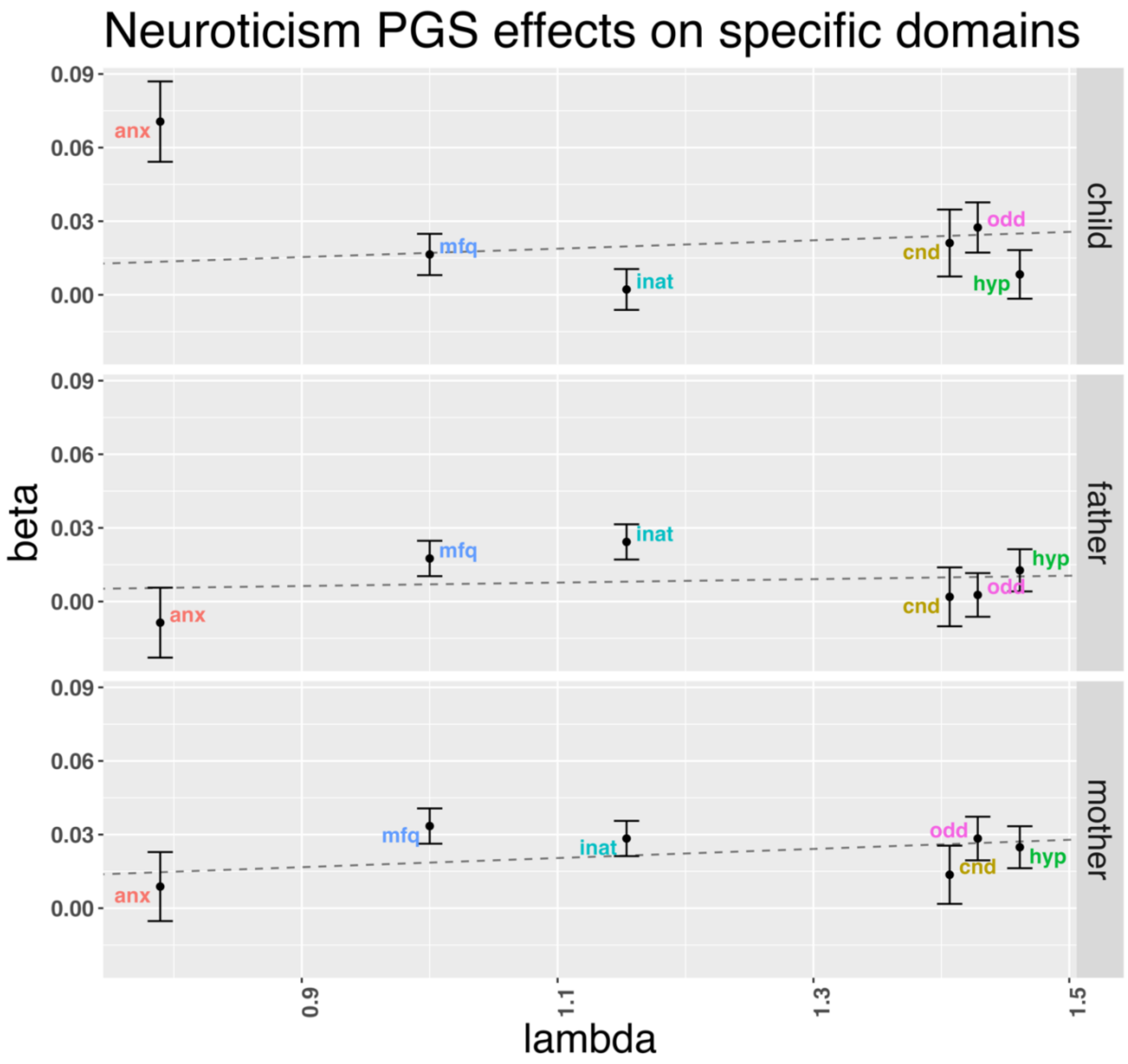

Figure S9. Parent-offspring PGS effects of Neuroticism over specific domains against item loadings for the general P domain. Sample size N = 14,959**.** Point estimates represent beta coefficients and error bars are 95% CIs.

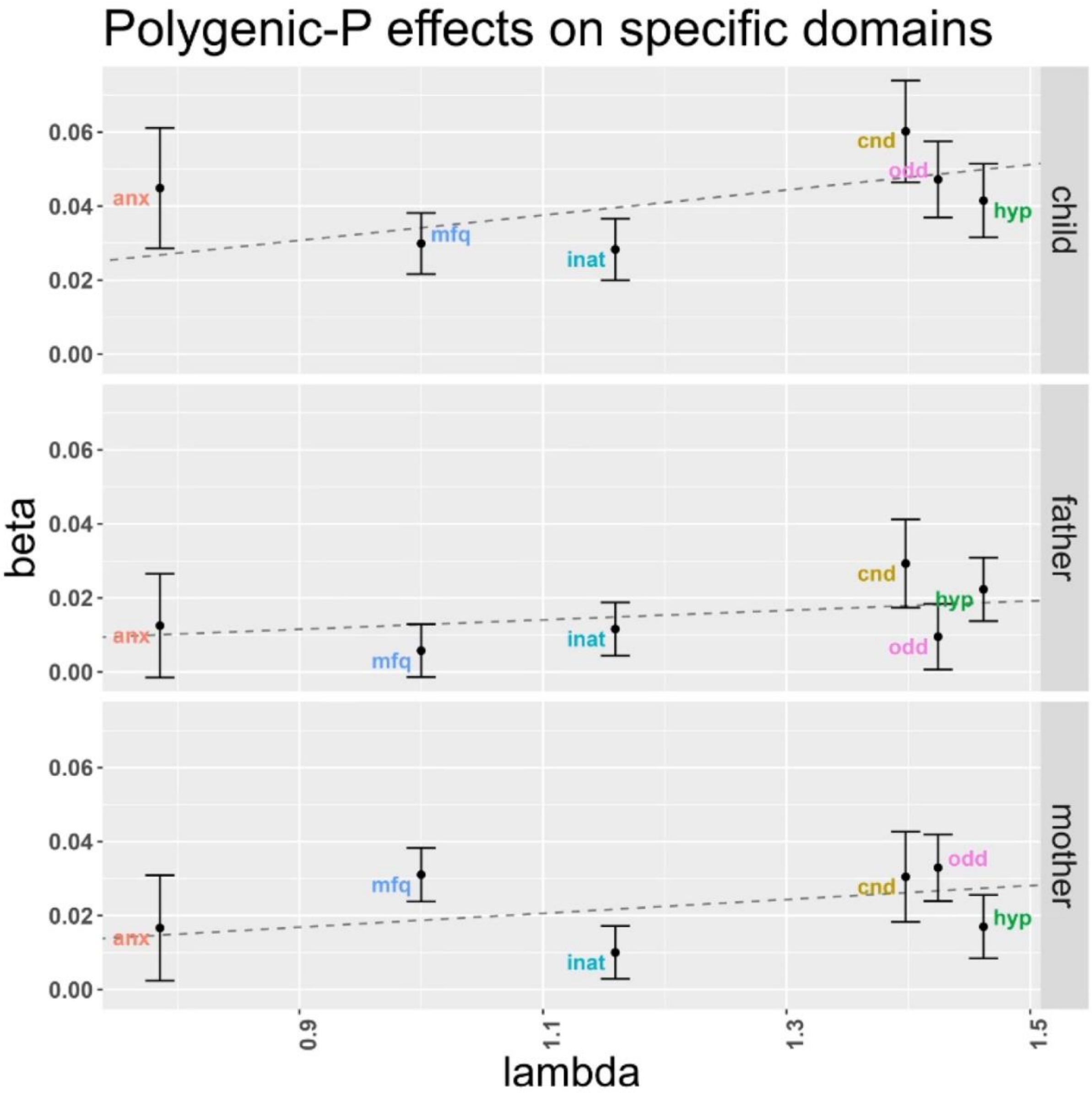

Figure S10. Parent-offspring PGS effects of polygenic-p over specific domains against item loadings for the general P domain. Sample size N = 14,959**.** Point estimates represent beta coefficients and error bars are 95% CIs.

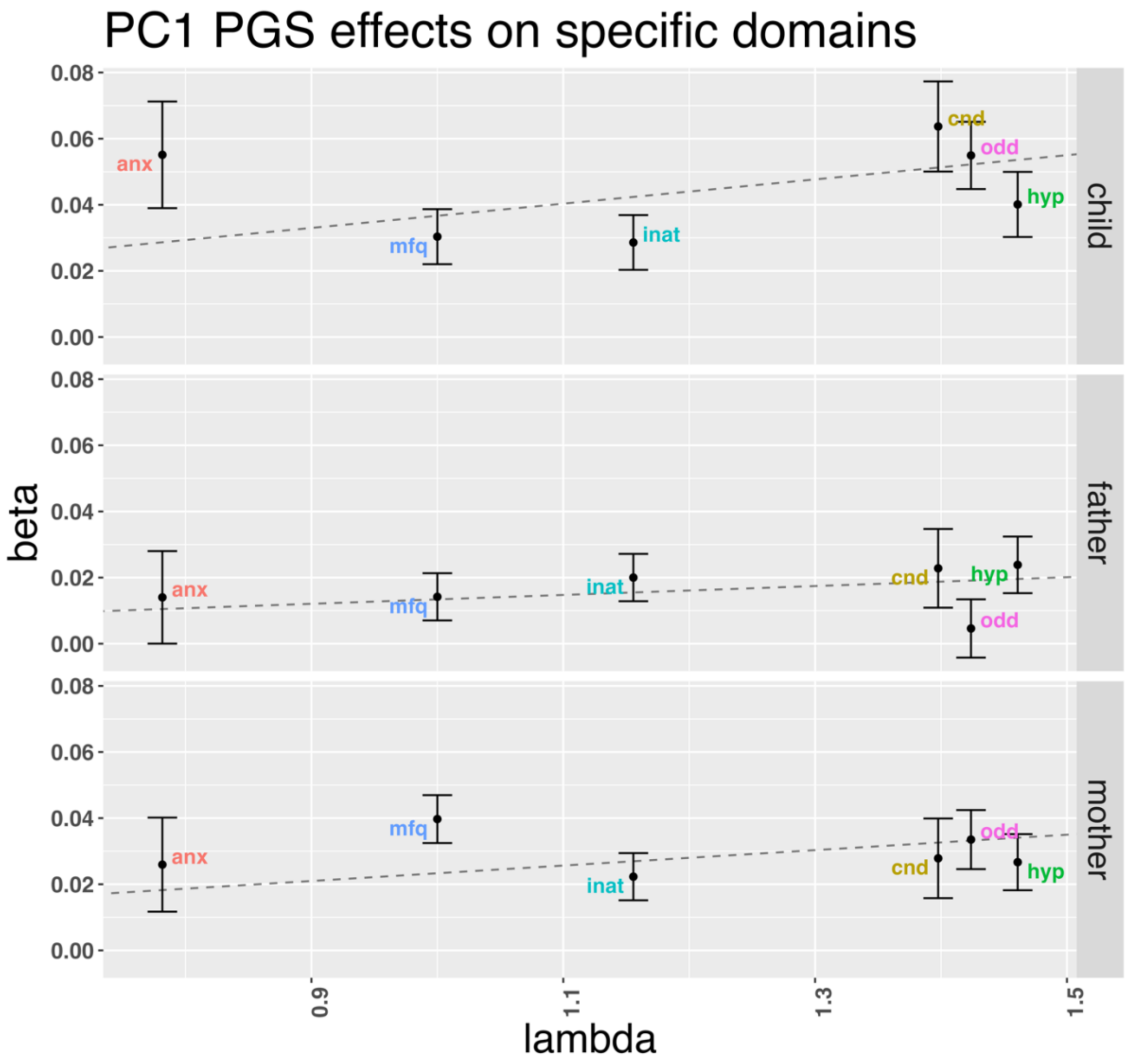

Figure S11. Parent-offspring PGS effects of the multivariate PC1 PGS over specific domains against item loadings for the general P domain. Sample size N = 14,959**.** Point estimates represent beta coefficients and error bars are 95% CIs.

References

1 Koch, T., Holtmann, J., Bohn, J. & Eid, M. Explaining general and specific factors in longitudinal, multimethod, and bifactor models: Some caveats and recommendations. *Psychological Methods* **23**, 505 (2018).

2 Heinrich, M. *et al.* On the meaning of the "P factor" in symmetrical Bifactor models of psychopathology: recommendations for future research From the Bifactor-(S− 1) perspective. *Assessment* **30**, 487-507 (2023).

3 Tubbs, J. D., Zhang, Y. D. & Sham, P. C. Intermediate confounding in trio relationships: The importance of complete data in effect size estimation. *Genetic Epidemiology* **44**, 395-399 (2020).

4 de la Fuente, J., Davies, G., Grotzinger, A. D., Tucker-Drob, E. M. & Deary, I. J. A general dimension of genetic sharing across diverse cognitive traits inferred from molecular data. *Nature Human Behaviour* **5**, 49-58 (2021).
