# Supplementary material for: Intergenerational transmission of polygenic predisposition for neuropsychiatric traits on emotional and behavioural difficulties in childhood
Source: Nat Commun. 2025 Mar 18;16:2674. doi: 10.1038/s41467-025-57694-w (PMC11920414; doi:10.1038/s41467-025-57694-w)
Supplement: Supplementary file 2 — Description of Additional Supplementary Files [file 41467_2025_57694_MOESM2_ESM.pdf]

Description of Additional Supplementary Files

Supplementary Data 1. Item frequencies

Supplementary Data 2a. Base models fit indices

Supplementary Data 2b. Base models comparison

Supplementary Data 3a. Standardized loadings - second-order model

Supplementary Data 3b. (Residual) variances - second-order model

Supplementary Data 3c. Standardized loadings - bifactor model

Supplementary Data 3d. (Residual) variances - bifactor model

Supplementary Data 4a. Polygenic-P PGS loadings – Child

Supplementary Data 4b. PC1 PGS loadings – Child

Supplementary Data 4c. Polygenic-P PGS loadings – Mother

Supplementary Data 4d. PC1 PGS loadings – Mother

Supplementary Data 4e. Polygenic-P PGS loadings – Father

Supplementary Data 4f. PC1 PGS loadings – Father

Supplementary Data 5. Model comparisons

Supplementary Data 6. Model comparisons - symptom heterogeneity

Supplementary Data 7. PGS results - trio models

Supplementary Data 8. PGS results – Child

Supplementary Data 9. PGS results – Parents

Supplementary Data 10. Shrinkage

Supplementary Data 11. Internal Consistency

Supplementary Data 12. PGS results - ADHD items trio models

Supplementary Data 13. PGS results - CPAIN items trio models

Supplementary Data 14. GWAS summary statistics
