# Supplementary material for: Intergenerational transmission of polygenic predisposition for neuropsychiatric traits on emotional and behavioural difficulties in childhood
Source: Nat Commun. 2025 Mar 18;16:2674. doi: 10.1038/s41467-025-57694-w (PMC11920414; doi:10.1038/s41467-025-57694-w)
Supplement: Supplementary file 4 — Reporting Summary [file 41467_2025_57694_MOESM4_ESM.pdf]

nature portfolio

Corresponding author(s): Andrea G. Allegrini

Last updated by author(s): 13/6/24

Reporting Summary

Nature Portfolio wishes to improve the reproducibility of the work that we publish. This form provides structure for consistency and transparency in reporting. For further information on Nature Portfolio policies, see our Editorial Policies and the Editorial Policy Checklist.

Statistics

For all statistical analyses, confirm that the following items are present in the figure legend, table legend, main text, or Methods section.

n/a	Confirmed	
☐	☒	The exact sample size (n) for each experimental group/condition, given as a discrete number and unit of measurement
☐	☒	A statement on whether measurements were taken from distinct samples or whether the same sample was measured repeatedly
☐	☒	The statistical test(s) used AND whether they are one- or two-sided<br>*Only common tests should be described solely by name; describe more complex techniques in the Methods section.*
☐	☒	A description of all covariates tested
☐	☒	A description of any assumptions or corrections, such as tests of normality and adjustment for multiple comparisons
☐	☒	A full description of the statistical parameters including central tendency (e.g. means) or other basic estimates (e.g. regression coefficient) AND variation (e.g. standard deviation) or associated estimates of uncertainty (e.g. confidence intervals)
☐	☒	For null hypothesis testing, the test statistic (e.g. F, t, r) with confidence intervals, effect sizes, degrees of freedom and P value noted<br>*Give P values as exact values whenever suitable.*
☒	☐	For Bayesian analysis, information on the choice of priors and Markov chain Monte Carlo settings
☒	☐	For hierarchical and complex designs, identification of the appropriate level for tests and full reporting of outcomes
☐	☒	Estimates of effect sizes (e.g. Cohen's d, Pearson's r), indicating how they were calculated

Our web collection on statistics for biologists contains articles on many of the points above.

Software and code

Policy information about availability of computer code

Data collection	*Provide a description of all commercial, open source and custom code used to collect the data in this study, specifying the version used OR state that no software was used.*
Data analysis	R 3.5, Lavaan 0.6-9, and LDPred2 (bigsnpr 1.12.16)

For manuscripts utilizing custom algorithms or software that are central to the research but not yet described in published literature, software must be made available to editors and reviewers. We strongly encourage code deposition in a community repository (e.g. GitHub). See the Nature Portfolio guidelines for submitting code & software for further information.

Data

Policy information about availability of data

All manuscripts must include a data availability statement. This statement should provide the following information, where applicable:

- Accession codes, unique identifiers, or web links for publicly available datasets
- A description of any restrictions on data availability
- For clinical datasets or third party data, please ensure that the statement adheres to our policy

The consent given by the participants does not open for storage of data on an individual level in repositories or journals. Researchers who want access to data sets for replication should submit an application to datatilgang@fhi.no. Access to data sets requires approval from The Regional Committee for Medical and Health Research Ethics in Norway and an agreement with MoBa.

Research involving human participants, their data, or biological material

Policy information about studies with human participants or human data. See also policy information about sex, gender (identity/presentation), and sexual orientation and race, ethnicity and racism.

Reporting on sex and gender	We focus our analyses on MoBA mothers, fathers and offspring genetic data, as well as the offspring phenotypes. Biological sex derived from chromosomal data was adjusted for in all analyses. Analyses were not run separately by sex because of power considerations.
Reporting on race, ethnicity, or other socially relevant groupings	Each model included fixed effects of child sex, year of birth, genotype chip, batch and 20 genetic principal components to control for the effects of these variables.
Population characteristics	We used data from version 12 of the quality assured MoBa data files. The genotype pipeline of the MoBa study is described in Corfield et al. (2022), which involved retaining only participants of European ancestry genotype data. Participants of this study were aged 8 years old, 51% were females.
Recruitment	Participants were recruited from MoBa a population-based pregnancy cohort study conducted by the Norwegian Institute of Public Health (Magnus et al., 2016; Paltiel et al., 2014). Participants were recruited from all over Norway from 1999-2008. The women consented to participation in 41% of the pregnancies. The cohort includes approximately 114.500 children, 95.200 mothers and 75.200 fathers. The current study is based on version 12 of the quality-assured data files released for research in January 2019.
Ethics oversight	The establishment of MoBa and initial data collection was based on a license from the Norwegian Data Protection Agency and approval from The Regional Committees for Medical and Health Research Ethics. The MoBa cohort is currently regulated by the Norwegian Health Registry Act. The current study was approved by The Regional Committees for Medical and Health Research Ethics (Ethical approval: 2016/1702)

Note that full information on the approval of the study protocol must also be provided in the manuscript.

Field-specific reporting

Please select the one below that is the best fit for your research. If you are not sure, read the appropriate sections before making your selection.

☐ Life sciences ☒ Behavioural & social sciences ☐ Ecological, evolutionary & environmental sciences

For a reference copy of the document with all sections, see nature.com/documents/nr-reporting-summary-flat.pdf

Behavioural & social sciences study design

All studies must disclose on these points even when the disclosure is negative.

Study description	Quantitative observational study.
Research sample	We used all available genotyped parent-offspring trios. The Moba sample is fairly representative, but genetic analyses were limited to individuals of European genetic ancestry. The impact of selection bias and attrition bias have been explored in previous studies (Biele et al., 2019; Nilsen et al., 2009). The final sample size was N = 14,959 genotyped family trios with at least one phenotypic observation available across emotional and behavioural difficulties when children were aged 8 years. 51% of children were female.
Sampling strategy	We used the largest sample of genotyped parent-offspring trios with mental health data available in MoBa. Power analyses showed that the sample was large enough to detect relevant effect sizes of interest.
Data collection	Phenotypic information was obtained via questionnaires completed by the mother or the father of the child collected when children were aged 8 years. Genetic data was obtained from blood samples from both parents during pregnancy and from mothers and children (umbilical cord) at birth.
Timing	MoBA has conducted many waves of data collection. We used parent-offspring trios with maternal reports from questionnaire data collected when children were aged 8 years, the first MoBa wave with an extensive range of measures for emotional and behavioural difficulties. Specifically, we used item-level data measuring symptoms of depression (Short Mood and Feelings Questionnaire; 13 items; Angold et al., 1995), anxiety (Screen for Child Anxiety Related Disorders; 5 items; Birmaher et al., 1999), conduct problems (18 items form the 31 items Rating Scale for Disruptive Behaviour Disorders [RS-DBD]; Silva et al., 2005), Oppositional defiant disorder (RS-DBD; 8 items), Hyperactivity (RS-DBD; 9 items) and Inattention (RS-DBD; 9 items).
Data exclusions	All participants were required to have genotype data available. Children were required to have symptom measures available. We used a threshold of .10 for the largest genetic correlation allowed between any two individuals (ignoring pairs of parents and offspring), to limit confounding due to closely related individuals being included in analyses.
Non-participation	The study is based on already collected data. No participant dropped out of the study.

Randomization	This was an observational study, no randomization was performed.

Reporting for specific materials, systems and methods

We require information from authors about some types of materials, experimental systems and methods used in many studies. Here, indicate whether each material, system or method listed is relevant to your study. If you are not sure if a list item applies to your research, read the appropriate section before selecting a response.

Materials & experimental systems

n/a	Involved in the study
☒	☐ Antibodies
☒	☐ Eukaryotic cell lines
☒	☐ Palaeontology and archaeology
☒	☐ Animals and other organisms
☒	☐ Clinical data
☒	☐ Dual use research of concern
☒	☐ Plants

Methods

n/a	Involved in the study
☒	☐ ChIP-seq
☒	☐ Flow cytometry
☒	☐ MRI-based neuroimaging

Plants

Seed stocks	*Report on the source of all seed stocks or other plant material used. If applicable, state the seed stock centre and catalogue number. If plant specimens were collected from the field, describe the collection location, date and sampling procedures.*
Novel plant genotypes	*Describe the methods by which all novel plant genotypes were produced. This includes those generated by transgenic approaches, gene editing, chemical/radiation-based mutagenesis and hybridization. For transgenic lines, describe the transformation method, the number of independent lines analyzed and the generation upon which experiments were performed. For gene-edited lines, describe the editor used, the endogenous sequence targeted for editing, the targeting guide RNA sequence (if applicable) and how the editor was applied.*
Authentication	*Describe any authentication procedures for each seed stock used or novel genotype generated. Describe any experiments used to assess the effect of a mutation and, where applicable, how potential secondary effects (e.g. second site T-DNA insertions, mosiacism, off-target gene editing) were examined.*
